# Is the association between physical activity and healthcare utilization affected by self-rated health and socio-economic factors?

**DOI:** 10.1186/s12889-015-2079-5

**Published:** 2015-08-01

**Authors:** Patricia Rocca, Anders Beckman, Eva Ekvall Hansson, Henrik Ohlsson

**Affiliations:** Department of Clinical Sciences in Malmö/General Practice, Lund University,, CRC, Jan Waldenströms gata 35:28:11, SE 205 02 Malmö, Sweden; Lund University Center for Primary Health Care Research, Malmö, Sweden

## Abstract

**Background:**

Physical activity and healthcare utilization has negative association. However, there appears to be limited knowledge of how this association is affected by self-rated health (SRH) and socio-economic status (SES). Therefore, the aim of this study was to examine the association between leisure-time physical activity (LTPA) and healthcare utilization, and investigate how SRH, gender, age and SES affected this association.

**Methods:**

A cross-sectional public health survey was conducted in Skåne, Sweden 2012, based on a random sample with 55,000 participants (response rate 51 %; 28,028 individuals included in the study) aged 18–80 years. The data was linked to individual healthcare utilization data and socio-economic data. Logistic regression analyses were conducted to study the association between LTPA and healthcare utilization. Path analysis was used to investigate the possible mediation effect of SRH to the association between LTPA and healthcare utilization.

**Results:**

Compared to sedentary leisure time the odds ratio for health care utilization decreased with increasing level of LPTA; physically active 0.89 (95 % CI: 0.81–0.96), for average exercise 0.74 (0.67–0.81) and for vigorous exercise 0.65 (0.60–0.72). The socio-economic variables attenuated this association to a small degree, but SRH had a strong impact. While the mediation analysis illustrated that the indirect effects were strong (and in the expected order so that higher levels of LTPA were more negatively associated with poor health) and highly significant, the direct effects suggested that higher levels of physical activity were more positively associated with healthcare utilization than lower levels. The indirect effects were substantially stronger than the direct effects.

**Conclusions:**

There was a significant negative association between decreased healthcare utilization and increased LPTA, and the association remained after adjustment for socio-economic variables. The mediation analysis (with SRH as the mediator between LTPA and healthcare utilization) showed that the indirect effects were strong and in the expected order, but the direct effects of LTPA on healthcare utilization was positive so that higher levels of LTPA had higher healthcare utilization. These results suggest that even though higher physical activity in total decreases the healthcare utilization, parts of the association that is not mediated through SRH actually increase healthcare utilization.

## Background

Physical inactivity is a major public health challenge in the developed world and recognized as a global epidemic [1]. The global estimate for prevalence of physical inactivity among adults is 17 % while 41 % have an insufficient level of physical activity [2]. In Sweden only half of the population achieves the recommended levels of physical activity. The other half has either a lower level of physical activity than recommended (40 %) or are physically inactive (10 %) [3]. A sedentary population implicates great economic consequences in terms of costs for healthcare and, indirectly, in costs for society in form of lost work force. The expenditure in Sweden for insufficient physical activity and low physical activity was 2002 estimated to 0.4 % respectively 3 % of the total costs in healthcare and production loss due to diseases [4].

Whether an individual strains physically or not has an effect on perceived health. Self-rated health (SRH) is a strong predictor of disease and mortality and has been shown to be a valid proxy for the current health state of the individual [5]. SRH measures are frequently applied in health research and reflect an individual’s attitude and beliefs of the biological, psychological and social dimensions of health [6, 7]. Previous research on physical activity and health-related quality of life implies that health-related quality of life tends to improve with increased physical activity, for all age groups [8, 9]. A study performed in European Union states that highly active or sufficient active individuals rated their health to be better than insufficiently active individuals. The same study presents a positive association between higher income, higher education and a good SRH [10]. Several studies confirm the relationship between physical inactivity, as well as poor SRH, and healthcare utilization. Individuals with low levels of physical activity or a sedentary lifestyle tend to use healthcare more often than active individuals [11, 12]. Even though several studies also show that socio-economic factors are associated with healthcare expenditure [13] there appears to be limited knowledge of how the association between physical activity and healthcare utilization is affected by SRH and socio-economic status (SES). Further, the concept of “objective” disease in relation to “subjective” illness (here SRH) cause difficulties in interpreting the stairway to contemporary heaven [14]. One way of controlling for disease is by using Adjusted Clinical Groups, ACG, which quantifies morbidity by age, gender and medical diagnoses [15].

The aim of this study was to examine the association between leisure-time physical activity (LTPA) and healthcare utilization in a Swedish population, and how this association is affected by SRH, gender, age, country of birth, educational status, individual disposable income and recorded disease. Our second aim was to investigate the possible mediating effect of SRH on the association between LTPA and healthcare utilization. Our hypothesis was that higher levels of LTPA are associated with SRH, which in turn predict less healthcare utilization.

## Methods

### Material

Linked register data from the Skåne Regional Council (individual healthcare utilization data and Adjusted Clinical Groups (ACG) and Resource Utilization Bands (RUB)) [16], Statistics Sweden (age, gender, education and individual income) and the public health survey in Skåne (Folkhälsa i Skåne 2012) was used. The linking was performed using the unique individual Swedish 10-digit personal ID number assigned at birth or immigration to all Swedish residents. This ID number was replaced by a serial number to preserve confidentiality.

### Study population

The study population was based on the public health survey that was conducted in 2012 in Skåne, which is the southernmost part of Sweden. The 2012 public health survey in Skåne is a cross-sectional study, and was sent to 55, 000 individuals from a random sample from the official population registers of people living in Skåne, who were born between 1932 and 1994. Two reminder letters were also administered to initial non-responders. In total 28,028 individuals responded to the survey, which represents approximately a 51 % participation rate.

### Ethics

All personal identifiers within the dataset were de-identified to ensure that individual information was fully protected and unknown to the authors. Ethical consent was obtained from the Ethical Review Board at Lund University, Sweden (No. 2014/199).

### Dependent variables

The public health survey was linked to individual healthcare utilization data managed by the Skåne Regional Council. The data included all registered individual visits in 2011 of the respondents to publicly funded physicians. Both primary and specialized care as well as public and private care was included. Visits due to preventive care, i.e. mainly child and maternity healthcare, were excluded. The outcome variable was then the dichotomised sum of all visits (i.e., yes versus no) irrespective of type of provider.

### Independent variables

From the public health survey the following variables were selected for the analyses:

- *Leisure-time physical activity (LTPA)* was based on the question “How much have you exercised and strained yourself physically the last 12 months in your leisure-time?” Four alternative responses were possible and the respondents answered by putting a cross in a box in front of the appropriate alternative:
  1. Regular physical activity and exercise (coded as vigorous exercise). You engage in for example running, swimming, tennis, badminton, keep-fit exercises at least 3 times per week for at least 30 min per time;
  2. Average, regular physical activity on your leisure-time (coded as average exercise). You are physically active 1–2 times per week for at least 30 min per time with running, swimming, tennis, badminton or other activity that makes you sweat;
  3. Average physical activity on your leisure-time (coded as physically active). You walk, bicycle or move yourself in other way for at least 2 h a week without sweating. Examples include walking and biking to and from work, other walks, gardening, fishing, table tennis, and bowling;
  4. Sedentary leisure-time (coded as sedentary). You engage mostly in reading, watching TV, going to the movies or other sedentary activities on your leisure-time. You walk, bike or move in other ways less than 2 h a week.

The coding was made in line with previous research [17]. In the analyses the sedentary group was used as reference.

*Self-rated health (SRH)* was assessed with the question “How do you value your current general health?” The optional answers were “Very good”, “Good”, “Average”, “Very poor” and “Poor”. “Very good” and “good” were collapsed to “good” and “very poor” and “poor” to “poor”. The coding was made in line with previous research [18]. In the analyses the group with good SRH was used as reference.

The public health survey was also linked to socio-economic data from Statistics Sweden. The following variables have been shown to be of importance in public health studies and were therefore used in the analyses [13, 18, 19]:

*Age* was stratified in age groups 18–29, 30–49, 50–64 and 65–80 years of age. In the analyses the age group 18–29 was used as reference.

*Gender* was categorized as males and females. In the analyses females were used as reference.

*Country of birth* was classified as Sweden, Other Scandinavian country, Other European country and Outside Europe. Country of birth was then dichotomized as Sweden-born or foreign-born. In the analyses foreign-born were used as reference.

*Educational status* was categorized into three groups based on the highest level of education within the family: low education (<= 9 years in school), middle education (10–12 years in school) and high education (more than 12 years). In the analyses high education was used as reference.

*Individual disposable income* was dichotomized in two groups in relation to the median income in the study population, i.e. income below median and income above median. In the analyses income above median was used as reference.

*Individual recorded disease* according to International Classification of Disease 10, ICD-10, was transformed to ACG and collapsed into RUB. The RUB is a six-level (0 - V) simplification of ACG, enhancing statistical analysis. The six levels range from non-health care users (=0) to very high health care users (=5). RUB was used in the analysis.

### Statistical analysis

For our first aim we used logistic regression to study the association between LTPA and healthcare utilization and how this association was affected by SRH, gender, age, country of birth, educational status and individual disposable income. In model A, only LTPA was included in the model. In model B1, LTPA and SRH were included in the model. In models B2-B6, LTPA and the socio-economic variables were included separately. Models B2-B6 was then also expanded by including an interaction term between the socio-economic variable and LTPA. Finally in model C, all variables were included in the same model. The results from the logistic regressions are presented as odds ratios (OR) with 95 % confidence interval (95 % CI). In order to investigate our second aim we conducted a mediation analysis. Using a path model with the probit link, we investigated the direct and indirect effect of LTPA (via SRH) on healthcare utilization. The estimates are presented as probit estimates with a 95 % CI. All analyses were performed using SPSS [20] version 22 and Mplus version 7.1 [21].

## Results

### Study population

In total 28,028 persons answered the questionnaire (51 % response rate). Table 1 show that the majority of the respondents were women (54.2 %), born in Sweden (84.3 %), with a middle (40.7 %) or high education (41.3 %), physically active (41.2 %), rated their health as good (71.5 %) and had utilised healthcare in 2011 (71.5 %). The mean age of the respondents was 51 years (StD: 16.9). A higher share of males had a sedentary lifestyle, but a higher share of females had poor SRH and utilised health care. Older individuals had to a higher degree than younger a sedentary lifestyle, poor SRH and a higher healthcare utilization. This pattern was seen for individuals with low SES as well. Individuals with poor SRH had to a higher degree a sedentary lifestyle and a higher healthcare utilization.

**Table 1:** Count (N), frequency (%) and total distributions (%) among 28,028 individuals included in the Skåne healah care survey, 2012

			LTPA				SRH			Healthcare utilisation	
Variable	Level	N (%)	Vigorous exercise	Average exercise	Physically active	Sedentary	Good	Average	Poor	Yes	No
Gender	Male	12,828 (45.8)	21.2	21.9	40.1	14.2	72.6	20.4	5.9	65.9	34.1
	Female	15,200 (54.2)	19.5	22.0	42.2	13.2	70.5	21.9	6.4	76.3	23.7
Age	18–29	4,035 (14.1)	30.2	26.6	30.9	10.6	83	13.3	3.3	57.2	42.8
	30–49	8,544 (30.5)	22.2	26.2	36.0	13.8	79.5	14.8	4.9	63.6	36.4
	50–64	7,912 (28.2)	16.8	20.7	45.4	14.4	67.4	23.1	8.7	73.9	26.1
	65–80	7,537 (26.9)	16.3	16.0	48.3	14.4	60.4	30.7	6.6	85.8	14.2
Country of birth	Sweden-born	23,641 (84.3)	20.9	22.8	41.9	12.2	72.6	21.2	5.2	72.5	27.5
	Foreign-born	4,372 (15.6)	16.7	17.5	37.8	21.2	65.3	21.4	11.6	66.3	33.7
Disposable income	Low	13,383 (47.7)	13.7	14.5	47.0	18.8	63.5	25.9	9.0	75.3	24.7
	High	13,389 (47.8)	18.4	18.5	42.7	16.3	78.7	17.1	3.4	70.5	29.5
Educational status	Low	4,103 (14.6)	10.3	10	17.2	21	57.7	30.8	9.4	79.6	20.4
	Middle	11,407 (40.7)	18.8	20.0	42.8	15.4	68.6	23.5	6.9	74.1	25.9
	High	11,567 (41.3)	23.9	26.4	38.1	10.0	78.9	15.9	4.4	69.2	30.8
LTPA	Vigorous exercise	5,671 (20.2)	-	-	-	-	86.3	10.9	2.2	67.0	33.0
	Average exercise	6,159 (22)	-	-	-	-	81.4	15.0	2.8	69.5	30.5
	Physically active	11,555 (41.2)	-	-	-	-	68.3	25.3	5.4	73.3	26.7
	Sedentary	3,826 (13.7)	-	-	-	-	47.7	32.5	18.6	75.6	24.4
SRH	Good	20,029 (71.5)	24.4	25.0	39.4	9.1	-	-	-	66.2	33.8
	Average	5,941 (21.2)	10.4	15.6	49.3	20.9	-	-	-	83.9	16.1
	Poor	1,736 (6.2)	7.0	9.9	35.7	41.1	-	-	-	88.7	11.3
Healthcare utilisation	Yes	20,049 (71.5)	23.5	23.5	38.7	11.7	84.8	12.0	2.5	-	-
	No	7,979 (28.5)	18.9	21.4	42.2	14.4	66.1	24.9	7.7	-	-

### Association between LTPA and healthcare utilization

Table 2, model A, show that the ORs for healthcare utilization decrease with increasing level of LTPA. Compared to sedentary leisure time the OR (95 % CI) for physically active was 0.89 (0.81–0.96), for average exercise 0.74 (0.67–0.81) and for vigorous exercise 0.65 (0.60–0.72). Model B1 show that SRH has a statistically significant impact on this association; the association disappears and is actually opposite for some groups, thus implying a strong impact. Note that SRH is statistically significant associated with healthcare utilization and that the association is strong with an OR of over 4 for poor compared to good SRH. The socio-economic variables attenuate the association between LTPA and healthcare utilization at a statistically non-significant degree (Model B2-B6). However, the individual socio-economic factors are statistically significant associated with healthcare utilization. In the full model (model C) the association between LTPA and healthcare utilization is absent.

**Table 2:** Results from the logistic regression. Numbers are Odds Ratios and 95 % Confidence intervals

		Model A	Model B1	Model B2	Model B3	Model B4	Model B5	Model B6	Model C
LTPA	Sedentary	1 (ref)	1 (ref)	1 (ref)	1 (ref)	1 (ref)	1 (ref)	1 (ref)	1 (ref)
	Physically active	0.89	1.11	0.87	0.84	0.86	0.88	0.87	0.92
		(0.81–0.96)	(1.01–1.21)	(0.80–0.95)	(0.77–0.92)	(0.79–0.93)	(0.80–0.96)	(0.79–0.95)	(0,83–1.02)
	Average exercise	0.74	1.03	0.73	0.83	0,71	0.77	0.74	1.03
		(0.67–0.81)	(0.94–1.14)	(0.66–0.80)	(0.75–0.91)	(0.65–0.78)	(0.70–0.85)	(0.67–0.82)	(0.92–1.14)
	Vigorous exercise	0.65	0.95	0.65	0.74	0.63	0.67	0.64	0.93
		(0.60–0.72)	(0.87–1.05)	(0.59–0.72)	(0.67–0.81)	(0.57–0.69)	(0.61–0.74)	(0.58–0.70)	(0.85–1.03)
SRH	Good	-	1 (ref)	-	-	-	-	-	1 (ref)
	Average	-	2.57	-	-	-	-	-	2.21
			(2.37–2.77)						(2.03–2.40)
	Poor	-	4.06	-	-	-	-	-	3.89
			(3.46–4.76)						(3.27–4.63)
Gender	Female	-	-	1 (ref)	-	-	-	-	1 (ref)
	Male	-	-	0.60	-	-	-	-	0,54
				(0.57–0.64)					(0,51–0,58)
Age	18–29	-	-	-	1 (ref)	-	-	-	1 (ref)
	30–49	-	-	-	1.29	-	-	-	1.23
					(1.20–1–40)				(1.12–1.35)
	50–64	-	-	-	2.04	-	-	-	1.77
					(1.88–2.21)				(1.60–1.96)
	65–80	-	-	-	4.34	-	-	-	3.81
					(3.96–4.77)				(3.43–4.24)
Country of birth	Foreign-born	-	-	-	-	1 (ref)	-	-	1 (ref)
	Sweden-born	-	-	-	-	1.39	-	-	1.17
						(1.29–1.49)			(1.08–1.28)
Educational status	High	-	-	-	-	-	1 (ref)	-	1 (ref)
	Middle	-	-	-	-	-	1.24	-	1.06
							(1.17–1.31)		(0.96–1.17)
	Low	-	-	-	-	-	1.62	-	1.14
							(1.48–1.77)		(1.07–1.21)
Disposable income	High	-	-	-	-	-	-	1 (ref)	1 (ref)
	Low	-	-	-	-	-	-	1.23	1.01
								(1.17–1.30)	(0.94–1.08)

Model A is adjusted for the variable LPTA, model B1 for SRH, model B2 for gender, model B3 for age, model B4 for country of birth, model B5 for educational status and model B6 for individual disposable income. In the full model (C) the association is adjusted for all variables

The next question we wanted to evaluate was weather there were any interaction effects between the socio-economic variables and LTPA, so that for example the effect of LTPA varied among different groups of SES. We assessed the inclusion of the interaction terms by evaluating if the model fit improved when including the interaction terms. These exploratory analyses suggested that the association between LTPA and healthcare utilization varied slightly between different age groups (p-value: 0.01), for different income groups (p-value 0.04), but not for gender (p-value: 0.13), for different groups of education (p-value: 0.33), for immigrant status (p-value: 0.20) or for SRH (p-value: 0.48). The significant interaction effects suggested while the OR for vigorous exercise was 0.93 in the youngest age group the OR was 0.60 for the age group 50–64; in other words the effect of the association between LTPA and healthcare utilization depended slightly on the age group you belonged to. Furthermore, the interaction analysis suggested that the OR for Average exercise was 0.82 for those with low income while the OR among those with high income was 0.68. These two interaction terms were the only significant interactions in the models.

**Fig. 1:**
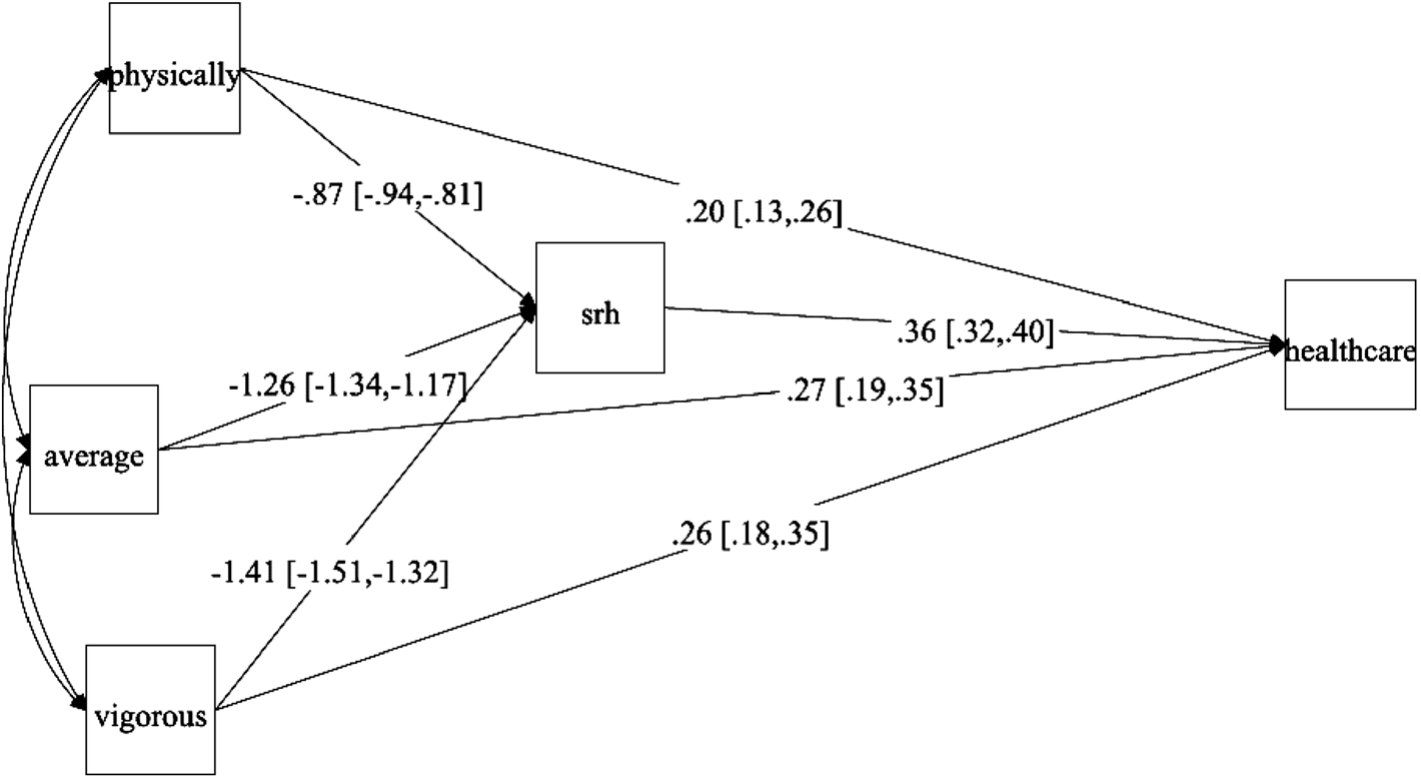
Path model with the probit link on 20,488 individuals from the Skåne public health survey. Path model with the probit link on 20,488 individuals from the Skåne public health survey. Physically = physically active; Average = Average active; Vigorous = Vigorous exercise; SRH = Self-rated health (coded as 0 = good health and 1 = Poor health); healthcare = Healthcare utilization (0: No and 1 = Yes)

The results from the path model are illustrated in Fig. 1. In order to simplify the models we excluded individuals that answered ‘average’ to the SRH question, so that the mediator became a binary variable (with poor health coded as 1 and good health coded as 0). As shown the association between different levels of physical activity was highly correlated with SRH and the effect sizes were in the expected order so that higher levels of LTPA were more negatively associated with poor health. We can also see that there is an expected positive association between poor health and healthcare utilization. The indirect effects (i.e. the effect from different levels of LTPA via SRH to healthcare utilization) are shown in Table 3 and they are all highly significant. The indirect effect (probit coefficients) from vigorous exercise (via SRH) to healthcare utilization is -0.51 (95 % CI: -0.57; -0.45; calculated by multiplying the path from vigorous exercise to SRH with the path from SRH to healthcare utilization), from average exercise -0.45 (-0.51; -0.40) and from physically active -0.32 (-0.35; -0.28). For the direct effects from different levels of LTPA to healthcare utilization we see some unexpected results. The direct effects from different levels of LTPA to healthcare utilization suggest that higher levels of physical activity are more positively associated than lower levels. Note however, that the indirect effects are substantially stronger than the direct effects. These results were only attenuated to a small degree when age, gender, income, education and immigrant status were included in the model (results not shown).

**Table 3:** Results from the Path model with the probit link on 20,488 individuals from the Skåne public health survey

	Total effect	Indirect effect	Direct effect
Physically active vs Sedentary	−0.12 (–0.18;–0.06)	–0.32 (–0.35;–0.28)	0.20 (0.13; 0.26)
Average active vs Sedentary	–0.18 (–0.25;–0.12)	–0.45 (–0.51;–0.40)	0.27 (0.19; 0.35)
Vigorous exercise vs Sedentary	–0.24 (0.31;–0.19)	–0.51 (–0.57;–0.45)	0.26 (0.18; 0.35)

### Sensitivity analysis

In sensitivity analysis where we excluded individuals with the two highest morbidity categories measured using the RUB (*N* = 2,308) the results were attenuated to some degree. In the empty model (Table 4) the OR for Vigorous exercise decreased from 0.65 to 0.72, and the OR for physically active decreased to 0.94. The path model showed the same pattern of results so that the associations were attenuated to some degree. For example the indirect effect (probit coefficients) from vigorous exercise (via SRH) to healthcare utilization is -0.42 (95 % CI: -0.48; -0.36), from average exercise -0.36 (-0.41; -0.31) and from physically active -0.26 (-0.300; -0.22).

**Table 4:** Odds-ratio (OR) and confidence intervals (95 % CI) for use of healthcare (excluding individuals with the two highest morbidity categories measured using the Resource Utilization Bands (RUB) *N* = 24,999

		Model A	Model B1	Model C
LTPA	Sedentary	1 (ref)	1 (ref)	1 (ref)
	Physically active	0.94	1.13	0.97
		(0.86–1.03)	(1.03–1.23)	(0.88–1.08)
	Average exercise	0.82	1.07	1.07
		(0.74–0.90)	(0.97–1.18)	(0.96–1.18)
	Vigorous exercise	0.72	0.98	0.97
		(0.65–0.79)	(0.89–1.08)	(0.88–1.08)
SRH	Good	-	1 (ref)	1 (ref)
	Average	-	2.33	2.04
			(2.15–2.52)	(1.88–2.22)
	Poor	-	3.45	3.08
			(2.84–3.95)	(2.58–3.68)

Model A is adjusted for the variable LPTA, model B1 for SRH,. In the full model (C) the association is adjusted for SRH, gender, age, education, income and country of birth

## Discussion

In this study we first replicated results from several previous studies [12, 22] showing that higher levels of LTPA were associated with decreasing healthcare utilization. This association was not attenuated by gender, age, country of birth, educational status or individual disposable income. These results suggest that individuals will, regardless of socio-economic characteristics, have a lower healthcare utilization with higher levels of LTPA. We also showed that the effect of LTPA varied slightly for some socio-economic variables. To notice was the fact that individuals in the age group 50–64 had a stronger effect of vigorous exercise on healthcare utilization than other age groups; this illustrate the importance of the continuation of physical activity. Furthermore, we show that the association between healthcare utilization and LTPA was strongly affected by SRH and disappeared when SRH was included in the models.

The association between healthcare utilization and LTPA and the affect SRH had on the association were more thoroughly investigated by a mediation analysis. This analysis showed that all the positive effects of LTPA on healthcare utilization were mediated through SRH. The only direct effect that we could estimate was opposite to what would have been expected, so that individuals with higher levels of LTPA had higher healthcare utilization. This type of mediation has been called competitive mediation and it has been suggested that such direct paths are often evidence of the effects of one or more omitted mediators. This might have a value for further theory building. In our case it could be the fact the individuals that are more conscious of their body and symptoms have higher healthcare utilization—and they are probably also more physically active; this could be an individual attitude of “the body as a temple”. We have found no previous studies investigating this aspect, and there is a call for other studies to try to replicate these findings and thereby find competing mediators of the effect of LTPA on healthcare utilization. On the other hand, it might be as simple as more vigorous physical activity increases the risk of injuries and thereby increase healthcare utilization. In earlier studies SRH has been shown to be strongly associated to use of physician services and intensity and amount of LTPA, with poor SRH resulting in more healthcare utilization and even small amounts of light-intensity LTPA related to a better SRH than no LTPA at all [23–25].

The pathways from LTPA to healthcare utilization are complex and diverse, but several studies have shown that inadequate level of physical activity, and thereby increased risk of injury, leads to higher healthcare expenditure [12, 22]. Still, our findings suggest that there might be some parts of the association that is mediated by an unknown factor and that actually increase healthcare expenditure. But, as our sample is cross-sectional we could not draw any inferences on causality. It is possible and even probable that healthier people are more prone to be physical active and not that physical activity leads to better health. It is also possible that people using the healthcare system don’t have the possibility to be physical active. As we had information on RUB we excluded those individuals in the two highest morbidity categories in order to exclude individuals that probably not were able to be physical active. These results showed that a part of the association probably was due to reverse causality, so that individuals with high healthcare utilization were unable to be physically active. Furthermore, one could assume that age would be a rather good proxy for health status, but the inclusion in the models did not attenuate the association between LTPA and healthcare utilization. Still, regardless of the pathways, it is of great interest for policy initiatives to understand that physical activity is associated with less healthcare utilization, but also that there might be a direct effect that actually increases the healthcare utilization. Unfortunately we do not have access to longitudinal data where these hypotheses or others such as aspects of attitude could be more thoroughly tested.

Although the response rate was only 51 %, the study population shows a good representation of the population in Skåne when comparing with figures from Statistics Sweden. The respondents were mainly women (54.2 %), Sweden-born (84.3 %), had a high education (41.3 %) and rated their health as good (71.5 %). The mean age of the respondents was 51 years. According to Statistics Sweden there were 82 % Swedish born in Skåne in the year 2012, the mean age was 41 years, 33 % had high education and 80.2 % rated their health as good [26]. The comparison shows that the study population was somewhat older and better educated than the general population in Skåne. However, although higher educational status has been shown to be associated to a better SRH, the respondents still had a lower frequency of good SRH than the general population in Skåne. It is, however, unlikely that response bias would explain the results obtained.

One limitation in studies of physical activity, and also in the present study, is that self-reported physical activity is difficult to measure, implying a risk for under—and overestimation of the level of LTPA. The question of LTPA used in this study does not exactly correspond to current recommendations, where adults aged 18–64 are recommended to perform at least 150 min of moderate-intensity aerobic physical activity throughout the week, or at least 75 min of vigorous-intensity aerobic physical activity throughout the week or an combination of both [2]. However, the questions correspond sufficient enough to rely on our results to be valid. Also, we had not access to data about occupational physical activity and it is possible that a higher amount of participants would have reached the recommended level of physical activity if occupational physical activity was included. Another limitation is that we have only used a broad measure of healthcare utilization; defined only as visiting or not visiting the healthcare system. As previous studies have shown and as we show in this study, is the fact that a majority of the population actually visit the healthcare system within a year.

## Conclusions

In conclusion, we replicate previous studies and show that there is an association between decreased healthcare utilization and increased LPTA. However, we also show that association remain after adjustment for socio-economic confounders and that the strength of the association varies with age. Finally we show in a mediation analysis (with SRH as the mediator between LTPA and healthcare utilization) that the indirect effects are strong and in the expected order, so that the effect of physical activity is mediated through SRH. But more importantly the direct effect of LTPA on healthcare utilization is positive so that higher levels of LTPA have higher healthcare utilization. These results suggest that even though higher physical activity in total decreases the healthcare utilization parts of the association that is not mediated through SRH actually increase the healthcare utilization. This can be a sign of an unidentified mediator that can be of interest for further theory building.
